# Small Molecule Inhibitors for Unc-51-like Autophagy-Activating Kinase Targeting Autophagy in Cancer

**DOI:** 10.3390/ijms24020953

**Published:** 2023-01-04

**Authors:** Ujjwala Karmacharya, Jong-Wha Jung

**Affiliations:** 1 College of Pharmacy, Research Institute of Pharmaceutical Sciences, Kyungpook National University, Daegu 41566, Republic of Korea; 2 Vessel-Organ Interaction Research Center, Kyungpook National University, Daegu 41566, Republic of Korea

**Keywords:** Unc-51-like autophagy-activating kinase, kinase inhibitor, autophagy, cancer

## Abstract

Autophagy is a cellular process that removes damaged components of cells and recycles them as biochemical building blocks. Autophagy can also be induced to protect cells in response to intra- and extracellular stresses, including damage to cellular components, nutrient deprivation, hypoxia, and pathogenic invasion. Dysregulation of autophagy has been attributed to various diseases. In particular, autophagy protects cancer cells by supporting tumor cell survival and the development of drug resistance. Understanding the pathophysiological mechanisms of autophagy in cancer has stimulated the research on discovery and development of specific inhibitors targeting various stages of autophagy. In recent years, Unc-51-like autophagy-activating kinase (ULK) inhibitors have become an attractive strategy to treat cancer. This review summarizes recent discoveries and developments in small-molecule ULK inhibitors and their potential as anticancer agents. We focused on structural features, interactions with binding sites, and biological effects of these inhibitors. Overall, this review will provide guidance for using ULK inhibitors as chemical probes for autophagy in various cancers and developing improved ULK inhibitors that would enhance therapeutic benefits in the clinic.

## 1. Introduction

Cells employ various mechanisms, including autophagy, for surviving during various environmental changes. Macroautophagy, generally referred to as autophagy, is a cellular process to remove damaged components of the cells and recycle them as biochemical building blocks. Autophagy can also be induced to protect cells in response to intra- and extracellular stresses, including damage to cellular components, nutrient deprivation, hypoxia, and pathogenic invasion [1]. The balance between autophagy activation and inhibition is regulated by signaling networks that allow cells to survive under various conditions.

Autophagy dysregulation has been observed in various diseases, including cancer, neurodegenerative disorders, as well as infectious and autoimmune diseases [1]. Thus, chemical modulation of autophagy has attracted increasing attention as a pharmacological intervention for these diseases. Among the molecular targets in autophagy signaling, Unc-51-like autophagy-activating kinase (ULK) family has attracted particular attention. The multi-protein complex of ULK is an upstream inducer of the core autophagy machinery [2]. It regulates autophagy at an early stage by serving as a starting point for recruiting the downstream components of autophagy. The ULK complex phosphorylates downstream proteins and is pivotal for initiating autophagy [3]. Therefore, ULKs have been suggested as promising molecular targets for developing novel therapeutic modalities [4]. Recently, a small molecule ULK1/2 inhibitor DCC-3116 was designed to inhibit autophagy in cancer cells and is under clinical trials in patients with advanced or metastatic solid tumors with RAS/mitogen activated protein kinase (MAPK) pathway mutation (ClinicalTrials.gov, NCT04892017, accessed on 20 December 2022). Other ULK1/2 inhibitors are still in the developmental stage, but a first-in-class, potent and selective anticancer agent is being pursued both in academic and industrial settings. This review summarizes the recent discoveries and developments in small-molecule ULK inhibitors and their potential as anticancer agents. It covers ULK1 and ULK2 inhibitors at development stage from scientific reports since 2015, focusing on the structural features, interactions with binding sites, and biological effects of various ULK inhibitors.

## 2. An Overview of ULKs in Autophagy and Cancers

Autophagy plays an important and dual role in tumor suppression and promotion in different contexts. The controversial aspects of autophagy in cancer have been reviewed and reported [5,6,7]. Autophagy removes damaged components of the cells and transfers harmful products to lysosomal degradation to prevent further cellular damage [8]. Autophagy also induces autophagic or programmed cell death to suppress cancer development [9]. Defects in autophagy can cause cellular damage to make genetically unstable cells, and initiate cancer development. Paradoxically, autophagy fulfills the high energy and metabolic demands of cancer cells and allows them to develop tolerance against stress [5]. Autophagy enables cancer cells to survive stresses such as energy deprivation and hypoxia [6]. Autophagy also enhances tumor metabolism and metastasis, thereby limiting chemotherapy [10]. Irrespective of the dual role of autophagy in cancer, however, growing evidence supports the theory that autophagy protects cancer cells by supporting tumor cell survival and drug resistance, and its inhibition has been suggested as a potential anticancer strategy [11,12,13].

**Figure 1:**
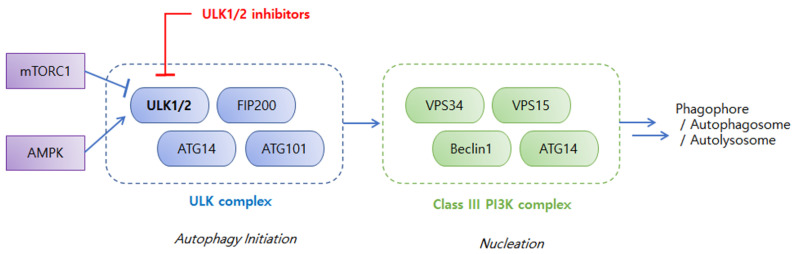
Early-stage regulations of the autophagy process. mTORC1 = mammalian or mechanistic target of rapamycin complex 1; AMPK = AMP-activated protein kinase; ULK1/2 = Unc-51-like autophagy activating kinase 1 or 2; FIP200 = focal adhesion kinase family-interacting protein of 200 kDa; ATG14 = autophagy-related protein 14; ATG101 = autophagy-related protein 101; VPS34 = vacuolar protein sorting 34; VPS15 = vacuolar protein sorting 15; PI3K = phosphoinositide-3-kinase.

Understanding the pathophysiological mechanisms of autophagy in cancer has fueled the research on discovery and development of specific inhibitors targeting various stages of autophagy [8,14,15]. The ULK complex is an early-stage regulator of autophagy and attracted particular attention as a drug target. Among ULK isoforms, ULK1, ULK2, ULK3, ULK4, and serine/threonine-protein kinase 36 (STK36), ULK1 have been most extensively studied. ULK1 is an evolutionarily conserved serine/threonine kinase [3] and forms a large ULK1 complex in human cells with autophagy-related proteins, such as autophagy-related protein 13 (ATG13), autophagy-related protein 101 (ATG101), and focal adhesion kinase family-interacting protein of 200 kDa (FIP200) [16]. As shown in Figure 1, the ULK1 complex is regulated by upstream kinases, mammalian or mechanistic target of rapamycin complex 1 (mTORC1), and AMP-activated protein kinase (AMPK), which sense cellular stresses to deliver the integrated input [17,18,19,20]. mTORC1 is a negative regulator of ULK1 complex. The ULK1 complex associates with active mTORC1 in a nutrient-rich environment to be phosphorylated at the inhibitory sites of ULK1 and ATG13, which inhibits the autophagy-promoting kinase activity of the ULK1 complex [19]. Dissociation of mTORC1 from the ULK1 complex during nutrition deprivation and cellular stress results in dephosphorylation of the inhibitory sites of the ULK1 complex and autophosphorylation at the active sites of ULK1, ATG13, ATG101, and FIP200, which triggers autophagy initiation [17]. mTORC1 suppresses the ULK1 complex under normal nutrient conditions whereas AMPK activates the ULK1 complex in a nutrition-deprived state. AMPK phosphorylates ULK1 at several sites and the activated ULK1 complex initiates autophagy by phosphorylating downstream regulators, such as vacuolar protein sorting 34 (VPS34) and Beclin1, in the Class III phosphoinositide-3-kinase (PI3K) complex, thereby inducing the cascade of autophagy processes, including phagophore nucleation and elongation, autophagosome formation, and fusion with lysosomes to form autophagolysosomes [2].

**Figure 2:**
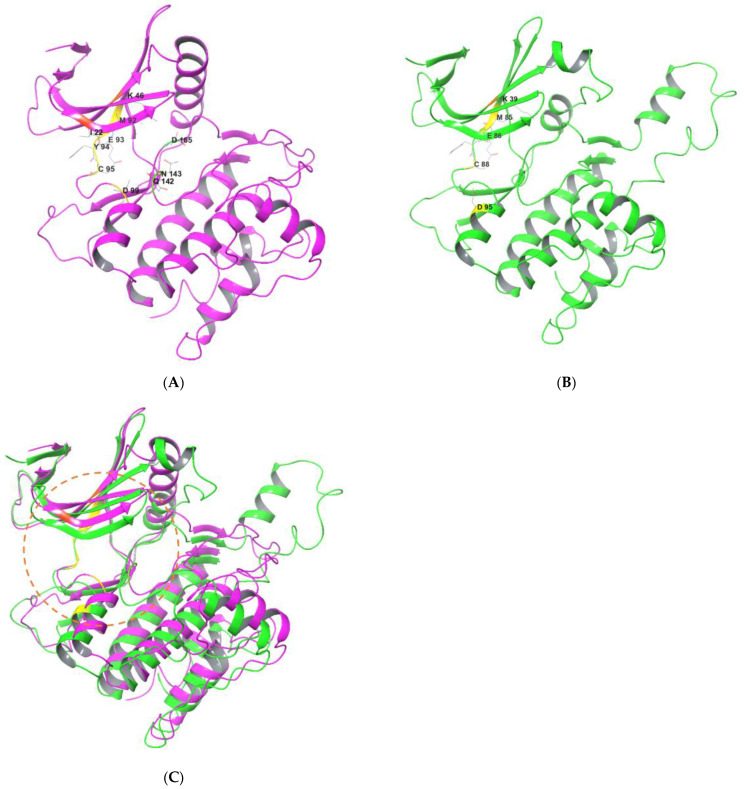
Structures of N-terminal kinase domain of ULK1 and ULK2. (**A**) Magenta: ULK1 KD ^a^ (PDB ID:6QAS). (**B**) Green: ULK2 KD ^a^ (PDB ID:6QAV). (**C**) Comparison of ULK1 and ULK2 KDs ^b^. ^a^ Key residues interacting with inhibitors are highlighted and labeled. Ligands and water molecules are omitted; ^b^ ULK1 and ULK2 KDs were superimposed with the Quick Aligh tool in Maestro 13.3 (Schrodinger Inc., New York, NY, USA). Orange circle: Binding site for ATP or inhibitors.

Figure 2 shows structural overviews of ULK1 and ULK2 kinase domains [21,22]. ULK1 possesses an N-terminal kinase domain (KD), a C-terminal domain (CD), and a serine-proline rich region between the KD and CD [6,18,20]. KD contains an adenosine triphosphate binding pocket between two lobes covered by a glycine rich loop. Figure 2A shows the structure of ULK1 KD (PDB ID:6QAS) [21,22]. The CD contains two tandem microtubule-interacting and transport domains that connect ULK1 with ATG13 and FIP200 to form the ULK complex. The region between KD and CD, which is rich in serine and proline, is a site for post-translational modification. It is a less conserved region that helps in substrate recognition and regulates kinase activity [18,20,23]. Figure 2B shows the structure of ULK2 KD (PDB:6QAV) [22]. ULK1 and ULK2 are similar in structure and share a high degree of domain architecture conservation. The entire protein structures of ULK1 and ULK2 show high similarity [20,24,25], allowing similar binding behaviors for inhibitors of both kinases (Figure 2C) [22]. In fact, most of the small-molecule inhibitors described in this review exhibit inhibitory activities against both ULK1 and ULK2.

Although the overall structures of ULK1 and ULK2 are highly superimposable, several structural and functional differences have been reported [20,22,26] in the arrangements of regulatory helix αC and activation segments. Furthermore, the β4-β5 and β6-β7 loop show conformational differences. ULK1 KD is also reported to be a typical monomeric structure, whereas ULK2 KD shows dimeric assembly. Both ULK1 and ULK2 are involved in autophagy induction and their functions are considered overlapping. However, growing evidence has highlighted functional differences between them. [20,26]

ULK3, ULK4, and STK36 are homologous to ULK1 and ULK2 only in the catalytic domain [27]. ULK3 contains N-terminal KD and microtubule interacting and trafficking (MIT) domains at C-terminus [28]. ULK3 is involved in the autophagy during senescence [29,30]. In resting state, N-terminal domain of ULK3 binds to suppressor of fused protein (SUFU) [31], and dissociation of ULK3 generates transcriptional activators, glioma-associated oncogene homolog zinc finger proteins (GLIs), key mediators in the Sonic hedgehog signaling [32]. ULK3-dependent GLI1 contributes to the transcriptional upregulation of DNA methyltransferase 3-alpha expression which is associated with autophagy induction [30]. ULK3 is upregulated in cancer-associated fibroblasts (CAFs) derived from head and neck squamous cell carcinomas, prostate cancer, and breast cancer. CAFs are under metabolic stress due to the increased demands for energy and nutrients, and which activates autophagy [33]. Because ULK3 is critical for CAF conversion for the pro-tumorigenic properties on neighboring cancer cells [34], it is a promising target for suppressing CAF activation and its tumor enhancement effects.

Crystal structure of ULK4 is available with ATPγS as well as its small molecule inhibitor [27,35]. ULK4 contains an N-terminal pseudokinase domain and repeated HEAT domains at C-terminus. Unlike other family members, the pseudokinase domain of ULK4 is catalytically inactive and has no phosphotranferase activity even though it can bind to ATP. Instead, the pseudokinase domain interact with other partner proteins. ULK4 is associated with blood pressure and neurodevelopment, and closely related to psychiatric disorders [36]. STK36 contains KD homologous to ULK1 and ULK2. STK36 interact with ULK4 for stable assembly of motile cilia, and it is required for cilia orientation in human respiratory epithelial cells [37]. The roles of ULK4 and STK36 in cancers are not clear yet.

## 3. Discovery and Development of ULK Inhibitors

**Figure 3:**
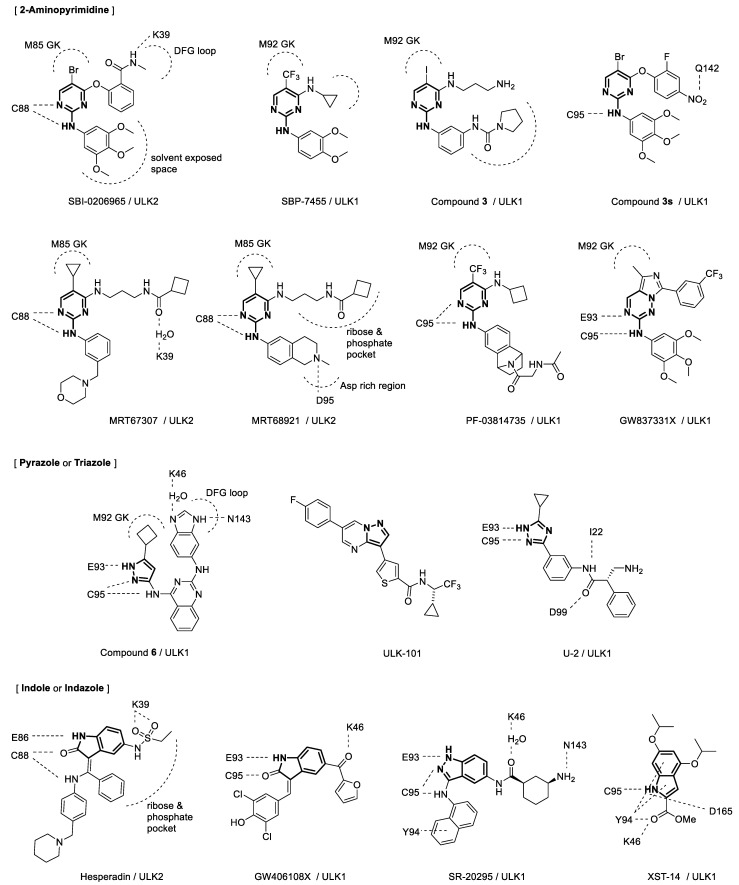
Structures of ULK1/2 inhibitors and key interactions with their binding sites. GK = gate keeper.

Functional, physiological, and structural studies on ULKs in autophagy processes have led to the discovery and development of ULK inhibitors. The structures of these small-molecule inhibitors are shown in Figure 3. Target-based screening of kinase-related chemical libraries in a reverse pharmacology approach was a popular method to discover ULK inhibitors possessing novel scaffolds of 2-aminopyrimidine, pyrazole, or indole. A chemical library was derived from a focal adhesion kinase (FAK) inhibitor exhibiting cross-reactivity toward ULK1, and in vitro ULK1 kinase screening of the library led to the discovery of a 2-aminopyrimidine compound, SBI-0206965 [38]. Although SBI-0206965 is a potent ULK1 inhibitor, its poor absorption after oral administration and poor systemic exposure following intraperitoneal dosing in mice has limited its use in vivo. To improve the potency and drug-like properties of SBI-0206965, a structure-based rational design was used to generate SBP-7455 [39]. Screening with a P-ATP radioactive assay revealed pyrazole aminoquinoline compounds as potent ULK1 inhibitors [21]. Remarkably, a selected pyrazole aminoquinoline derivative dramatically stabilized the ULK1 KD, which aided the crystallization of ULK1. Using the ligand-bound crystal structure, compound 6 possessing pyrazole aminoquinoline scaffold was developed as the most potent ULK1 inhibitor among the series. Although compound 6 had high affinity in inhibition of ULK1, it’s activity was not specific to ULK1, and it was not suitable for cellular use. Thus, screening continued for another lead compound, and compound 3 was identified as a potent and selective ULK1 inhibitor for further development [40]. MRT67307 and MRT68921, initially synthesized as TBK1 inhibitors possessing 2-aminopyrimidine scaffold, were also discovered as potent inhibitors for both ULK1 and ULK2. Screening of a kinase-focused library using differential scanning fluorimetry revealed Aurora A kinase inhibitors, such as PF-03814735 and hesperidin, as potent binders for both ULK1 and ULK2 [22]. ULK1 activity screening led to the discovery of pyrazolopyrimidine compounds including ULK-101 [18]. GSK Published Kinase Inhibitor Sets were screened for ULK inhibition, and GW837331X and GW406108X were identified as potent ULK1 inhibitors [41].

Virtual screening methods also have been employed for the discovery of ULK inhibitors. Docking-based pharmacophore modeling led to the discovery of 2-aminopyrimidine compound 3s, as a potent ULK1 inhibitor [42]. In silico high-throughput screening, combined with a biochemical assay, identified an indazole compound as an ULK inhibitor [43]. Further optimization led to the development of SR-20295 with nanomolar potency. Structure-based virtual docking with visual inspection and in silico filtering for drug-like properties highlighted an indole compound as a potent ULK1 inhibitor including XST-14 [44]. Pharmacophore-based virtual screening combined with structure-based docking led to the development of U-2, which exhibited a sub-micromolar IC_50_ value against ULK1 [45].

## 4. Binding Interactions between ULK1/2 and Their Inhibitors

Key interactions between ULK inhibitors and the binding sites in ULK1 or ULK2 proteins are presented in Figure 3 focusing on the structures of inhibitors. The crystal structures of ULK1 with PF-03814735 and of ULK2 with hesperidin were determined, and the characteristics of the ligand binding sites were then determined based on these structures [22]. Inhibitors exhibited typical type I kinase interactions as they bound to the active conformations of ULK1 and ULK2 in the ATP pockets. Flexible methionine GK and two unusually large binding pockets within the ribose- and phosphate-binding pockets allow plasticity of the kinase catalytic domain.

Crystal structure analysis of the ULK2 KD with SBI-0206965 revealed the following pharmacophores and key interactions [39]: 1-N and 2-N of the pyrimidine ring formed H-bonds with the hinge region, the amide substituent extended toward the DFG loop, and the aniline moiety was directed toward the solvent-exposed space. Other 2-aminopyrimidine compounds have similar interactions with their targets. 5-trifluoromethyl group of SBP-7455 provides additional interactions for insertion in the back pocket compared to those provided by the bromo group in SBI-0206965 while the 4-cyclopropylamine moiety of pyrimidine formed hydrophobic interactions [39]. These interactions might be attributed to the improved potency and binding affinity of SBP-7455 for both ULK1 and ULK2 compared with those of SBI-0206965 and other derivatives. A structure-activity relationship study and structural analysis of protein-ligand interactions also suggested that the pyrrolidine urea moiety of compound 3 contributed to the high selectivity for ULK1 by occupying space in the pocket [21]. Compound 3s is structurally related to SBI-0206965, but molecular docking studies with ULK1 have revealed small differences between the two; H-bonding between the hinge region and Q142, along with hydrophobic segments, contribute to the stronger binding of compound 3s to ULK1, which is beneficial for improving ULK1 inhibitory activity [42]. Structural analysis of the crystal structures of MRT67307 and MRT68921 bound to ULK2 suggested that the distinct interaction between the benzopiperidine of MRT68921 and the aspartate-rich region is important for the higher affinity of MRT68921 than that of MRT67307 [22]. Interestingly, H-bonding between cyclobutylamide of MRT67307 and K39 is mediated by a water molecule at the binding site. Docking poses for GW837331X was proposed with an established hinge binding with E93 and C95, and GW837331X presents a methyl group directing a pocket adjacent to the methionine GK [41].

Crystal structure of ULK1 KD with compound 6 revealed that compound 6 binds to the ATP-binding site, making hinge contacts with its aminopyrazole, while the cyclopropyl fits into a pocket adjacent to the gatekeeper (GK) methionine [21]. The binding also induces conformational changes in the kinase domain, and the DFG motif accommodates benzimidazole. H-bonding between benzimidazole and K46 is mediated by a water molecule at the binding site as in the case of MRT67307 and ULK2, while H-bonding with N143 is also observed for benzimidazole. The binding pose of U-2 with ULK1 was evaluated from molecular docking [45]. U-2 possessed a triazole scaffold and showed a similar hinge binding pose to compound 6 possessing an aminopyrazole scaffold. In addition, it showed an extended structure interacting with the hinge and I22, and the extended conformation aids in interaction with multiple amino acids in the kinase domain of ULK1.

Docking analysis of SR-20295 suggested that the aromatic system of naphthalene and amino group near the hinge contributed improved potency [43]. The aromatic system can engage in a pi interaction with Tyr94 of ULK1, while the amino group provides an additional H-bond with the hinge. The H-bonding with K46 is mediated by a water molecule similar to that in compound 6. Furthermore, the stereochemistry of cyclohexane is important for H-bonding with N143 [43]. XST-14 strongly bound to the full-length ULK1 protein and specifically bound to ULK1 KD as determined by surface plasmon resonance (SPR) experiments [44]. The key interactions between XST-14 and ULK1 KD included H-bonds with K46, Y94, and D165, as evidenced by the predicted docking poses and diminished binding of XST-14 to mutated ULK1 proteins in SPR analyses. H-bonds with the C94 hinge and π-stacking with Y94 were also observed. Docking poses for GW406108X was proposed with an established hinge binding with E93 and C95, and GW406108X has a carbonyl group interacting with K46 above the GK [44].

## 5. Biological and Anticancer Effects of ULK Inhibitors

Biological effects of ULK inhibitors including anticancer effects against specific cell lines are summarized in Table 1. SBI-0206965 is one of the most extensively explored ULK1 inhibitors and a potential anticancer agent [38]. SBI-0206965 is a potent inhibitor for both ULK1 and ULK2. SBI-0206965 suppresses mTOR kinase inhibitor-induced ULK1-dependent autophagy in A549 lung cancer cells. It also promotes apoptosis via inducing the loss of autophagic maintenance involved in cancer cell survival [38]. Furthermore, SBI-0206965 suppresses cell growth and promotes apoptosis in neuroblastoma cell lines [46]. SBI-0206965 triggers apoptosis in clear cell renal cell carcinoma cells by upregulating ULK1 mRNA expression and inhibiting xenograft tumor growth in mice [47]. SBI-0206965 induces caspase-dependent apoptosis in FLT3-ITD-mutated acute myeloid leukemia (AML) cells by inhibiting autophagy and increasing the reactive oxygen species levels [25]. Although SBI-0206965 is a potent ULK1 inhibitor, its poor absorption after oral administration and poor systemic exposure following intraperitoneal dosing in mice has limited its use in vivo [39].

SBP-7455 exhibited improved potency and binding affinity for both ULK1 and ULK2 compared with those of SBI-0206965 [39]. SBP-7455 significantly reduced ULK1-dependent Beclin1 and VPS34 phosphorylation, and inhibited autophagy in cells. Importantly, SBP-7455 demonstrated improved drug-like properties, such as improved physicochemical and pharmacokinetic profiles. After per oral administration of SBP-7455, downregulation of total ULK1 and ATG13 levels, as well as inhibition of ATG13 phosphorylation by ULK1, were observed in mouse liver samples [39]. Notably, SBP-7455 effectively reduced the viability of triple-negative breast cancer (TNBC) cells [39]. Furthermore, it induced high cytotoxicity in TNBC cells and increased apoptosis under starvation conditions. Moreover, it inhibited autophagic flux and decreased the number of autophagosomes.

Compound 6 is a highly potent ULK1 inhibitor with a single digit nanomolar IC_50_, but it was not suitable for cellular use because its activity was not specific to ULK1 [21]. Compound 3 is not as potent as compound 6, but it is a potent and selective ULK1 inhibitor which enabled further development in cells [40]. Compound 3 inhibited autophagy in cells via ULK1, as evidenced by the accumulation of LC3-I relative to that of LC3-II, a common marker of autophagosome formation.

MRT68921 exhibited nanomolar IC_50_ values for both ULK1 and ULK2 [24]. It also suppressed autophagy in MEF cells in a ULK1-dependent manner. MRT68921 exerts cytotoxic effects in various cancer cell lines and has a comparatively high safety window [48]. MRT68921 induced caspase-dependent apoptosis in FLT3-ITD-mutated AML cells by inhibiting autophagy and increasing reactive oxygen species, similar to SBI-0206965 [25]. Furthermore, MRT68921 diminished the c-AMP-mediated protective effect on the survival of DNA damage-induced B cell precursor acute lymphoblastic leukemia cells [49]. It also suppressed autophagy and viability by inhibiting ULK1 in high-grade serous ovarian cancer spheroids [50]. 

Compound 3s exhibited stronger ULK1 inhibitory activity at 10 µM than that of SBI-0206965 [42]. On treatment of 3s, autophagy substrate p62 increased while conversion of LC3 I to LC3II reduced together with reduction in Beclin1. i.e., compound 3s effectively blocked autophagy by inhibiting ULK1. The anti-proliferative activity of compound 3s was the most prominent in A549 lung cancer cells among five different cell lines tested, including leukemia and breast cancer cells.

PF-03814735 and hesperidin exhibited nanomolar K_D_ values for both ULK1 and ULK2 in isothermal calorimetry experiments [22]. The anticancer effects of PF-03814735 and hesperidin were reported before they were identified as ULK inhibitors, and the relationship between their anticancer effects and ULK inhibitory activities remains unclear. PF-03814735 was originally developed as an Aurora A kinase A/B inhibitor and has completed a phase I clinical trial for advanced solid tumors [51,52]. Hesperidin is a known natural product with anticancer activity [53].

SR-20295 is a potent ULK1 inhibitor with an excellent stability against drug metabolism [43]. It was quite stable in human, rat, and mouse microsomes, and exhibited negligible CYP inhibition. However, further studies including its effects against ULK dependent cancers has not been disclosed yet.

ULK-101 exhibited nanomolar inhibitory activity for both ULK1 and ULK2 [18]. ULK-101 suppressed mTOR inhibitor-induced formation of early autophagic vesicles, omegasomes, and phagophores. ULK-101 also reduced both basal and induced autophagy. Moreover, ULK-101 reduced the survival of osteosarcoma cells in starvation media compared to that in full growth media. A KRAS mutant or amplified non-small cell lung cancer (NSCLC) cells also showed increased ULK-101 sensitivity in starvation medium. Thus, ULK-101 is suggested to suppress autophagy-dependent cancer cell survival.

XST-14 exhibited nanomolar IC_50_ for ULK1 [44]. XST-14 also inhibited ULK2, almost as effectively as ULK1. XST-14 strongly inhibited the conversion of LC3-I to LC3-II and blocked autophagosome/lysosome fusion in CHO cells, indicating a blockade of the autophagic flux. XST-14 decreased the number of LC3 puncta and autophagic vacuole volume density in starved HepG2 cells. XST-14 also inhibited the phosphorylation of downstream targets of ULK1, PIK3C3, and Beclin1, decreased their interaction with ULK1, and destabilized the PIK3C3 and Beclin1 complex in HepG2 cells subjected to starvation-induced autophagy. XST-14 decreased the proliferation and invasion of hepatocellular carcinoma cells and induced apoptosis in a ULK1-dependent manner.

GW837331X and GW406108X exhibited sub-micromolar IC_50_s for ULK1 [41]. Enzyme kinetic studies revealed that the inhibitory effects of the compounds were primarily attributed to competitive inhibition of ATP to ULK1. Both compounds also inhibited ATG13 phosphorylation via ULK1 kinase activity and blocked the autophagic flux induced by amino acid starvation in cells. GW837331X and GW406108X were also reported to inhibit ULK2 with similar activities against ULK1 [41].

U-2 exhibited a sub-micromolar IC_50_ value against ULK1 [45]. U-2 decreased the expression level of LC3-II after co-treatment with the lysosomotropic agent chloroquine in HeLa cells under starvation conditions, indicating the blockade of autophagic flux in these cells. U-2 also showed selective cytotoxicity in human liver cancer cells compared to that in normal liver cells. In silico ADMET predicted that the compound U-2 possesses good drug-like properties; however, no experimental evidence has yet been provided for in vitro or in vivo ADME.

**Table 1:** ULK1/2 inhibitors as potential anticancer agents.

	Compound	IC_50_ (ULK1)	IC_50_ (ULK2)	Cancer (Cell Line)	Synergistic Co-Treatment
1	SBI-0206965	108 nM [38] 306 nM [22] 130 nM [39]	711 nM [38] 3.88 μM [22]	Non-small cell lung cancer (A549, H460) [38,54] Neuroblastoma (SK-N-AS, SH-SY5Y, SK-N-DZ) [46] Renal cell carcinoma (A498, ACHN) [47] Leukemia (MV4-11, MOLM-13, HL-60, U937) [25,55] Breast cancer (MDA-MB-231, MCF-7) [56]	mTOR inhibitor AZD8055 [38], daunorubicin [55], doxorubicin [56], cisplatin [54], TRAIL ^a^ [46]
2	SBP-7455	13 nM [39]		Triple negative breast cancer (MDA-MB-468, BT549, MDA-MB-231) [39]	PARP inhibitors olaparib, niraparib [39]
3	Compound 6	8 nM [21]		-	
4	Compound 3	120 nM [40]	360 nM [40]	-	
5	MRT67307	45 nM [24] 170 nM [40] 38.2 nM [22]	38 nM [24] 230 nM [40] 92.3 nM [22]	Leukemia (THP-1, U939, Molt4, HEL92.1.7, K562, Raji, Jurkat, HL60) [57]	
6	MRT68921	2.9 nM [24] 17.0 nM [22]	1.1 nM [24] 20.8 nM [22]	Mesothelioma (M28, REN) [58] ^b^ Leukemia (REH, MV4-11, MOLM-13, HL-60, U937, THP-1, Molt4, HEL92.1.7, K562, Raji, Jurkat, HL60) [25,49,57] Ovarian cancer (OVCAR3/4/8, COV318/362, CaOV3) [50] Various tumors [48]	Carboplatin and pemetrexed [58]
7	Compound 3s	99.15% inhibition at 10 μM [42] ^c^		Lung cancer (A549) [42] Lymphoma (U937) [42] Breast cancer (HL60) [42] Acute myeloid leukemia (MDA-MB-469) [42]	
8	PF-03814735	K_D_ 18.1 nM [22] ^d^	K_D_ 58.0 nM [22] ^d^	Solid tumors [51,52]	
9	Hesperidin	K_D_ 16.8 nM [22] ^d^	K_D_ 47.3 nM [22] ^d^	Various tumors [53]	
10	SR-20295	45 nM [43]		-	
11	ULK-101	8.3 nM [18]	30 nM [18]	Osteosarcoma (U2OS) [18] Non-small cell lung cancer (H838, H727, H2030, A549) [18]	
12	XST-14	13.6 nM [44]	70.9 nM [44]	Hepatocellular carcinoma (HepG2, Hep3B) [44]	sorafenib [44]
13	GW837331X	646 nM [41]	Similar to ULK1 ^e^ [41]	-	
14	GW406108X	427 nM [41]	Similar to ULK1 ^e^ [41]	-	
15	U-2	0.5 μM [45]		Hepatocellular carcinoma (SMMC-7721, HepG2, L02) [45]	

^a^ TNF-related apoptosis inducing ligand; ^b^ Multicellular spheroids; ^c^ IC_50_ was not determined; ^d^ Binding affinity from isothermal calorimetry experiments; ^e^ Similar inhibition of ULK1 (IC_50_ or quantitative inhibitory activity was not reported).

## 6. Selectivity Issues in ULK Inhibitors

Even highly selective ULK inhibitors can inhibit a range of additional kinases. Some ULK inhibitors were originally discovered as other kinase inhibitors and their ULK inhibitory activities were recognized later. Therefore, the anticancer effects are likely due to the cumulative effects of multiple target modulations; the inhibitors may also exhibit multiple off-target effects. Experimental evidence has been reported for some early ULK inhibitors.

SBI-0206965 inhibited 10 out of 456 purified human kinases in a competition-binding assay at 10 µM although SBI-0206965 was especially selective for ULK1 compared to other top binding kinases. SBI-0206965 appeared to be selective toward FAK in vitro and in vivo, possibly because its structure was derived from a FAK inhibitor; however, this selectivity was not comparable to that for ULK1 [38]. Furthermore, SBI-0206965 was identified as a direct inhibitor of AMPK, an upstream regulator of the ULK complex [59]. Notably, other kinases, including AMPK-related kinases, were reported to be inhibited by SBI-0206965; NUAK1 and MLK1, in particular, were comparably or more potently inhibited by SBI-0206965 than AMPK or ULK1 [60]. SBI-0206965 was also reported as a non-specific inhibitor of the AMPK/ULK-signaling in skeletal muscles [61]. Compound 3s also exhibited inhibitory activity against other kinases including AMPKα1 and FAK [42].

MRT68921 inhibited 44 kinases out of 80 tested kinases at 1 µM concentration [24]. In particular, MRT68921 inhibited TBK1 and AMPK kinases which are known to play pivotal role in autophagy. Notably, MRT68921 was able to inhibit autophagy in TBK1 or AMPK pathway independent manners. MRT68921 also inhibited NUAK1, a critical component of the antioxidant defense system [48].

ULK1 inhibitors, including SBI-0206965, MRT67307, and MRT68921, strongly interact with Aurora A, suggesting that Aurora A kinase is a major off-target substrate of ULK1/2 inhibitors [22]. Even though MRT68921 exhibited a significant anti-tumor effect in vitro and in vivo in leukemia cells, the effect was not considered relevant to both autophagy and reactive oxygen species [57]. Both SBI-0206965 and MRT68921 induced spindle microtubule disorganization and inhibited the phosphorylation of histone H3 in tumor cells [62]. ULK-101 inhibited 4 out of 327 recombinant human kinases [18], and XST-14 inhibited 6 out of 403 kinases in competitive binding assays [44]. GW837331X and GW406108X did not affect the upstream regulatory kinases, mTORC1 and AMPK, but they displayed promiscuous kinase selectivity profiles implying that other kinases may also regulate autophagosome formation [63].

## 7. Combination Therapy of ULK Inhibitors with Other Anticancer Agents

Drug induced autophagy protects cancer cells by supporting their survival, and which develop resistance against anticancer therapy [13]. Thus, exploiting ULK inhibitors to inhibit cytoprotective autophagy is a promising therapeutic strategy not only as a monotherapy but also as a combination of other anticancer agents. Notably, Deciphera’s ULK1/2 inhibitor, DCC-3116, is in clinical trial with RAS/MAPK pathway mutation (ClinicalTrials.gov, NCT04892017, accessed on 20 December 2022), because treatment of RAS mutant cancer cells with MAPK pathway inhibitors increases autophagy for cancer cell survival [64].

Synergistic treatment of developmental stage ULK inhibitors in combination of anticancer agents are presented in Table 1. Co-treatment of SBI-0206965 and mTOR inhibitor AZD8055 synergistically increased apoptosis up to 23% in lung cancer cells, while treatments of AZD8055 resulted in 7% apoptosis [38]. SBI-0206965 inhibited cell growth by 10% in the study. SBI-0206965 also enhanced cytotoxicity of topoisomerase inhibitors, daunorubicin and doxorubicin. It enhanced the cytotoxicity of daunorubicin in AML cells by inhibiting drug-induced autophagy via the AMPK/ULK1 pathway [55]. Besides, SBI-0206965 slowed down cell growth and promotes cell death in doxorubicin-resistant breast cancer cells [56]. SBI-0206965 also inhibited the proliferation of NSCLC cells, and sensitized them to cisplatin by inhibiting cisplatin-induced cancer cell-protective autophagy [54]. SBI-0206965 also sensitized neuroblastoma cells to TNF-related apoptosis-inducing ligand (TRAIL) treatment [46]. Interestingly, the study reported that SBI-0206965 cannot sensitize neuroblastoma cells to mTOR or topoisomerase inhibitors, INK128, Torin 1, doxorubicin or topotecan, which contrasts with the findings of other reports [38,56]; thus, these contrasting results needs further validation.

SBP-7455 potentiated the cytotoxic effect of poly(ADP-ribose) polymerase inhibitor (PARP) on TNBC cells by suppressing the drug-induced increase in autophagic flux [39]. Treatment of SBP-7455 with PARP inhibitors, olaparib or niraparib exhibited strong anti-proliferative synergy. SBP-7455 reversed olaparib-induced upregulation of autophagic flux in TNBC cells and intensified the cytotoxic effect of olaparib [39].

MRT68921 reduced the autophagic flux in multicellular spheroids generated from mesothelioma cell line M28 and potentiated the chemosensitivity to carboplatin and pemetrexed treatments [58]. MRT68921 significantly increased apoptosis in multicellular spheroids with high level of autophagy, compared to the chemotherapy alone. Notably, autophagy at steady state are in good correlation with chemosensitivity, and late stage autophagy inhibitor hydroxychloroquine does not potentiate chemosensitivity [58].

The combination of XST-14 and sorafenib, a multi-kinase inhibitor, synergistically suppressed the proliferation and invasion of hepatocellular carcinoma cells and reduced tumor weights and volumes in tumor xenograft mice [44]. Notably, XST-14 has an ester group that is rapidly hydrolyzed to carboxylic acid in vivo [44], suggesting that the metabolite is active.

## 8. Conclusions

Autophagy protects cancer cells by supporting tumor cell survival and drug resistance. Early studies on modulating autophagy focused on drug repositioning of indirect autophagy inhibitors and combination therapy. Recent developments in identifying small-molecule inhibitors against specific autophagy processes have highlighted ULK inhibitors as key anti-cancer agents and probes to interrogate the relationship between autophagy and cancer. However, some challenges still hinder the application of ULK inhibitors in cancer treatment. Multitarget involvement of ULK inhibitors when exerting anti-cancer effects often hinder the interpretation of in vivo outcomes. Most small molecules targeting kinases show cross-activity with other kinases, possibly owing to the structural similarities in their binding sites, and ULK inhibitors are no exception. In addition, ULK1 and ULK2 are highly similar in structure, particularly for the ligand-binding sites in KD; thus, achieving selective inhibition of ULK1 or ULK2 is challenging. Furthermore, hurdles in absorption, distribution, metabolism, and excretion of the molecules also need to be overcome for their effective in vivo application. Membrane permeability, non-specific protein binding, metabolic stability, and active transporters or efflux pumps often limit the in vivo applications of ULK inhibitors and may be responsible for their inconsistent in vivo application. Thus, developing improved ULK inhibitors is still desirable.

Overall, this review will be helpful in guiding the development and design of improved ULK inhibitors. Although the promiscuous nature of ULK inhibitors raises issues of selectivity and multiple targets, their anticancer effects are arguably significant and at least partially attributable to the inhibition of cytoprotective autophagy. Therefore, the discovery and development of autophagy-specific ULK inhibitors, as well as the comprehensive characterization of their biological activities, are essential to provide their anticancer therapeutic benefits in clinical settings.

## Data Availability

Not applicable.
